# New Appliances and Things Medical

**Published:** 1904-06-25

**Authors:** 


### NEW APPLIANCES AND THINGS MEDICAL.

[We shall be glad to receive, at our Office, 28 & 29 Southampton Street, Strand, London, W.C., from the manufacturers, specimens of all new preparations and appliances which may be brought out from time to time.]

### NEKTAR WINES.

(Reinheimer and Co., Surbiton, Surrey.)

The samples of these wines we have received (half-bottles) are as follows: Burgundy (red), Schlossberg (white), Apple (white), Tokayer, and Cider. They contain practically no alcohol, at least under 2 per cent., and are made from unfermented grape juice. This grape juice is prevented from fermentation by being sterilised. The wines contain no preservative, but they will not keep for any length of time after being opened. These beverages all contain a relatively large amount of grape and fruit sugar—15 to 25 per cent.; their acidity is considerable, and they contain a relatively large amount of malates and tartrates, and hence are somewhat laxative in their action. As far as we have been able to ascertain, they are quite wholesome and refreshing drinks. Too much importance should, however, not be attached to the name given to the individual member of the series. Since the bouquet-giving substances of the ordinary wines, which characterise them as individuals, are by-products of fermentation, it is obvious that they cannot be generated in these wines, in which no fermentation is allowed to take place.

